# Are physiotherapists and occupational therapists following the guidelines for discharge summary?–An analysis of the content of physiotherapists’ and occupational therapists’ discharge summaries and their adherence to stroke guideline recommendations

**DOI:** 10.1371/journal.pone.0308039

**Published:** 2024-09-03

**Authors:** Liss Marita Solbakken, Antje Sundseth, Birgitta Langhammer, Therese Brovold

**Affiliations:** 1 Department of Rehabilitation Science and Health Technology, Oslo Metropolitan University, Oslo, Norway; 2 Department of Neurology, Akershus University Hospital, Nordbyhagen, Lørenskog, Norway; University of Pretoria, SOUTH AFRICA

## Abstract

**Purpose:**

Discharge summaries are important tools for communication between health care levels and can ensure continuity of rehabilitation. This study aims to gain insight into the content of discharge summaries written by hospital physiotherapists and occupational therapists regarding patients with stroke, and their adherence to recommended criteria for discharge summaries.

**Material and methods:**

31 physiotherapy and multidisciplinary discharge summaries, for stroke patients discharged home from hospital with need of follow-up, were included in the study. We employed qualitative content analysis and descriptive statistics to explore and describe the content.

**Results:**

The physiotherapists and occupational therapists adhered to the recommended criteria for content in varying degree. The main focus for physiotherapists and occupational therapists were description of ADL, sensorimotor and general cognitive functions, they rarely report tolerance to exercise, and the specific cognitive abilities to follow instruction and learn were often omitted. Less focus was put on patients’ experiences and needs during acute stroke, and description of goals were omitted in the physiotherapy discharge summaries.

**Conclusion:**

While the physiotherapists and occupational therapists complement each other in their assessment of patients and inform the reader about both sensorimotor and cognitive functions and abilities, they omit some of the specific criteria for rehabilitation. Despite the omissions, the information provided is specific to the patients’ function and needs.

## Introduction

To ensure continuity of rehabilitation after stroke, the hospitals should convey patients’ medical history and rehabilitation needs to the primary healthcare in a discharge summary [1, 2]. Physiotherapists (PTs) and occupational therapists (OTs), along with other members of the multidisciplinary team (MDT) at the hospital, assess the patient’s functions and impairments, and how this affects the patient’s ability to perform activities (ICF) [3] before initiating acute care and rehabilitation [4]. Their discharge summary can also act as referral to further follow-up or rehabilitation in the primary healthcare [1, 2, 5], thereby being a tool for informational continuity and impact the patients’ follow-up [6].

A high-quality discharge summary should include relevant and necessary information about the patient’s personal and environmental factors, as well as medical information and initial care, to secure suitable follow-up in a timely manner [1, 5, 7]. This is supported and included in various stroke guidelines [2, 4, 8–10]. Studies report that high-quality information and collaboration between healthcare professionals across organization can enhance patients’ safety [11], improve knowledge and decision-making [12, 13] and lead to continuity of follow-up in the primary healthcare [14, 15].

However, a recent systematic review from Sheehan et al. [16] concludes that there is no universally accepted method of communication between allied health professionals in hospital and primary healthcare. They highlight the need to develop multidisciplinary communication to facilitate seamless transitions of care. The review further emphasizes that effective communication and information transfer to primary healthcare require both communication and collaboration across organizations and with the patients. However, research reports ineffective communication between healthcare settings when information about current treatment and further needs is lacking, especially when patients need further rehabilitation after discharge [17]. Multiple studies report that hospital doctors tend to omit both description of the stroke patient’s functions [17, 18] and allied health professionals reports and recommendations [16–19] in their discharge summaries. As input from PT and OT is important for recommending rehabilitation in the primary healthcare [20], their discharge summaries become essential in ensuring that patients’ rehabilitation needs are adequately conveyed and effectively transitioned to subsequent therapists.

OT and PT documentation in the patients record regarding patients with neurological diagnosis tend to be comprehensive, with focus on impairments and activity [21, 22]. Discharge information from hospital PTs and OTs are appreciated by therapists in the primary healthcare [14], and often guide assessment and interventions [23]. Nevertheless, primary healthcare PTs have expressed concern. While they appreciate advice on follow-up, they do not want strong recommendations from the hospital’s therapists [23]. Recommendations from the hospital tend to create expectations among patients and their families which may be challenging to fulfill due to resource constraints [12, 23].

As with other professions, it seems to be a challenge to align the understanding of what constitutes relevant information between the therapists in hospital and primary healthcare. To support communication between levels of care, the Norwegian stroke care pathway has established specific must- and should-criteria, Table 1 [2]. These criteria outline the functions and needs that should be addressed by the hospitals’ MDT to help the primary healthcare identifying patients who would benefit from the various rehabilitation services available in the municipality. Considering the varying understanding of what constitute relevant information, and little is known of the PT and OT discharge information delivered to the subsequent therapist in the primary healthcare, this qualitative study aims to gain insights from the content of discharge summaries written by the hospital PTs and OTs. The aims of this study are twofold:

1. To explore how hospital PTs and OTs present patients’ functions and needs in their discharge summaries, with a particular focus on patient’s involvement, their function and treatment, and the incorporation of the must- and should-criteria.
2. To describe the extent to which the discharge summaries adhere to the guideline’s recommendations.

**Table 1:** Recommended must- and should-criteria from the stroke care pathway.

Must-criteria, description of the patients’:	• Degree of disability/dependence (in activities of daily living, ADL) • Ability to understand instructions • Learning ability—cognitive ability to remember from time to time • Motivation—self-effort
Should-criteria, description of the patients’:	• Disabilities and/or functional impairments (sensorimotor and cognitive function) • Function prior to the stroke • Comorbidity/morbidity • Toleration of high intensity of exercise • Concrete and realistic goals • Rapid improvement of neurological impairment/disability

## Material and methods

### Study design

This qualitative study is part of a project on transitional care for patients with acute stroke (TracStroke) [24]. The overarching aim of TracStroke is to explore the ways in which discharge summaries influence continuity of follow-up among patients discharged from the hospital after a stroke, inspired by pragmatism to understand experiences, actions and consequences [25].

The project followed the ethical principles developed by the Declaration of Helsinki [26, 27], and was assessed by National Research Ethics Committee (no. 160207), and approved by the Norwegian Social Science Data Service (no. 925964) and the University Hospital’s data protection official (no. 20/09104). Furthermore, all study participants gave their written informed consent, which included consent for publications of the study’s findings.

### Setting

In Norway, the majority of patients with acute stroke is treated in stroke units and the median length of hospital stay is five days [28]. About 35% of the patients require rehabilitation after discharge, and 59% of them are referred for rehabilitation in the primary healthcare. Discharge summaries are transferred electronically via a secure national health net to the primary healthcare, and the summaries from the hospital PTs and OTs are referral for follow-up by the primary healthcare PTs and OTs. In addition to being guided by the stroke care pathway, the transfer of information from hospital PTs and OTs is also mandated by the Norwegian Health Personnel Act, which requires them to provide necessary and relevant documentation regarding the healthcare they have delivered to subsequent therapists [29]. The Norwegian stroke care pathway’s checklist includes key information of the medical status, personal, and environmental factors, as well as results of assessments. The must- and should-criteria specify important information as these are directly linked to expected rehabilitation benefits and are thought to ensure correct level of rehabilitation after discharge from hospital [2]. Most of these criteria aligns with the focus of the PTs’ and OTs’ assessment and are therefore relevant for them to report.

### Recruitment

The participants of this study were recruited from the stroke and in-patient neurological rehabilitation units at a large urban university hospital in Norway from January 1^st^ 2021 to September 30^th^ 2022 before their discharge. The criteria for participation in the current study were;

1. Diagnosis of acute stroke
2. Discharged home
3. The need for further follow-up by either PT or OT, as described in the discharge summaries by the hospital OT, PT, or MDT.

Patients were not included if they were unable to understand and speak Norwegian over the telephone or scored below 20 on the Mini Mental State Examination (MMSE) cognitive assessment tool [30].

The nurses at the stroke and rehabilitation units informed potential patients about the study, both in writing and verbally. Subsequently, the nurses informed the first author about the interested participants, who then provided additional verbal and written information about the study before written consent was obtained. All participants were informed that their participation was voluntary and that it would not influence their rehabilitation at either the hospital or the primary healthcare. Written consent was granted by 34 participants. Unfortunately, it turned out that four patients did not meet the inclusion criteria, as two had MMSE <20, one was discharged to a nursing home instead of their home, and one was diagnosed with transient ischemic attack rather than a stroke. These four were excluded from the study. Thus, the study included 30 patients and, as one patient had both a PT and MDT discharge summary, 31 discharge summaries.

### Data collection and analysis

The first author retrieved the participants’ discharge summaries from the hospital’s electronic records. After details of the participants and authors were anonymized, the discharge summaries were stored and analyzed in services for sensitive data [31].

The analysis was inspired by qualitative content analysis described by Schreier’s [32], where a coding frame is used to analyze the material. As qualitative content analysis allows quantification of data within the coding frame’s categories [32–34], it enabled us to address both aims of the study. NVivo 12 software was used for this analysis. The premise for conducting this analysis was the research question—What information is provided by PTs and OTs in stroke patients discharge summaries, especially regarding patients’ involvement, their function and treatment? The first and last author developed, piloted, and modified the coding frame, before the main coding was conducted by the first author. Each step of the analysis was discussed with the other authors. In developing the coding frame, the first and last authors discussed their preconceptions and were attentive to these when reading and coding the data. The development of the preliminary coding frame was guided by the two dimensions included in the research question noted above—patient involvement, and function and treatment. The analytical process was initiated by developing data-driven categories and sub-categories from six discharge summaries. Thereafter, the coding frame was expanded using deductive categories based on the pathway’s recommendations and was piloted. Furthermore, the coding frame’s subcategories were examined and described to meet the required unidimensional, mutually exclusive, and exhaustive standards, Table 2. Lastly, the main coding of all the data was conducted by the first author using the modified coding frame, Table 3.

**Table 2:** Examples of second-level subcategory descriptions guiding the coding and analysis.

Second-level sub-category	Description of categories, with example quotes from the discharge summary
Motivation	Description of the patients’ wishes, participation in treatment, or lack of motivation—e.g., *the patient is motivated for individual follow-up*
Goals	Measurable, achievable, relevance to activity/profession—e.g., *the patient wants to walk independently*
Cognitive assessments	Impairment level, descriptions of memory, processing skills, planning, ability to follow instructions, learning ability, clarity, and orientation—e.g., *patient exhibiting reduced short-term memory*
Sensorimotor assessments	Description of impairment level, including facial palsy, fine motor deficit, sensory deficits, strength, pace, coordination, and balance—e.g., *possible slight failure when walking*, *left-sided hemiparesis*

**Table 3:** The coding frame—categories and first level sub-categories.

Categories with subcategories	
**The patient in focus** • Patient’s experience of functions and abilities • Goals and wishes • Motivation • Participation in society • Family as a resource	**The patient’s outcome and functions after stroke** • Observations of activities • Cognitive assessment • Sensorimotor assessment • Assessment tools • OT and PT interventions

To describe the extent to which the discharge summaries adhere to the pathway’s recommendations, the sub-categories obtained from the qualitative content analysis, Table 3, that aligned with the pathway criteria were considered variables. Furthermore, descriptive statistics, median (min, max) and frequency were used for the description of these findings.

The coding frame and results were presented to and discussed with a user group including a user from a stroke patient organization, and PTs and OTs working in hospital and primary healthcare, to verify a clear understanding of the categories and their descriptions.

## Results

### Description of the participants and their discharge summaries

The study included 30 patients and 31 discharge summaries. The characteristics of the participants included in this study are shown in Table 4. Among the 31 discharge summaries, 19 were PT discharge summaries and 12 were MDT discharge summaries consisting of contributions from both the PTs and OTs. None of the participants had discharge summaries written exclusively by an OT. Across the discharge summaries most participants exhibited sensorimotor symptoms. Dizziness and problems with balance were observed to be acute symptoms in a third of the participants, while two experienced confusion. Furthermore, the median MMSE score of the 10 assessed participants was 25, while the median Short Physical Performance Battery [35] score of the 18 assessed participants was 9, indicating some mobility limitations [36].

**Table 4:** Description of participants.

Participant description	Participants N = 30
Gender: n, male/female	15/15
Age: y, median (min–max)	76 (46–95)
Married: n	16
Still working: n	9
Stroke: n, ischemic/hemorrhagic	28/2
Length of stay in stroke unit: d, median (min–max)	7 (1–11)
Length of stay in rehabilitation unit: d, median (min–max)	18.5 (12–31)
Need for further treatment after discharge: n, PT/OT/both	24/2/4
Discharge summary from: n, PT/OT/MDT	19/0/12

The stroke unit and rehabilitation unit from which the participants were recruited followed different templates for their discharge summaries. In the stroke unit, each occupation wrote their own discharge summaries for their colleagues in the primary healthcare when participants needed follow-up after their discharge. However, the PTs’ discharge summaries included medical information that was copied from the doctors’ reports in the participants’ journal. Meanwhile, the rehabilitation unit wrote MDT discharge summaries.

### The PTs’ and OTs’ adherence to the pathway’s must and should criteria

The discharge summaries examined in this study adhered in varying degrees to the recommendations offered by the pathway. The must- and should-criteria, presented in Table 5, exhibit the differences between the PTs discharge summaries in the stroke unit and the PTs and OTs notes in the MDT discharge summaries.

**Table 5:** The discharge summaries adherence to the must- and should-criteria.

Criteria	PT DS^1^, N = 19	MDT DS, N = 12	
		PT	OT
**Must-criteria**			
Activities of daily living, n (%)	19 (100)	12 (100)	11 (92)
Understanding instructions, n (%)	1 (5)	4 (33)	2 (17)
Learning ability, n (%)	0	2 (17)	2 (17)
Motivation, n (%)	2 (11)	7 (58)	4 (33)
**Should-criteria**			
Comorbidity^2^, n (%)	18 (95)	12 (100)	12 (100)
Prior function^2^, n (%)	19 (100)	12 (100)	12 (100)
Goals^2^, n (%)	1 (5)	12 (100)	12 (100)
Sensorimotor function, n (%)	16 (84)	11 (92)	6 (50)
Cognitive function, n (%)	1 (5)	0	9 (75)
Intensity	1 (5)	1 (8)	0
Improvement	10 (53)	8 (67)	2 (17)

^1^ discharge summaries

^2^ Reported by the MDT or copied from the medical records

### PTs’ and OTs’ descriptions of patients’ functions and needs

This section presents the qualitative results of PTs and OTs descriptions of patients’ function and needs in the PT and MDT discharge summaries. It describes the qualitative categories, Table 3, and the ways in which some of the criteria, Table 5, were incorporated into the discharge summaries.

#### The patients in focus

The patients’ experiences of their own functions and abilities were closely linked to the descriptions of their goals and their motivation. The patients’ voices were especially evident in descriptions of challenges they experienced in their functioning and activities post-stroke in comparison to their prior function. In some instances, patients’ prior functions and activities were reported in the descriptions pertaining to their goals and interventions.

*Quotes from MDT discharge summary 15* –*Main goal: returning home. Secondary goals: better balance and strength in the right leg, execute kitchen activities with the OT.*

The goals were often shortly described. The primary goals for rehabilitation were often described based on the ICF’s participatory levels, such as returning home and managing ADL. Most secondary goals were described at the activity level, such as walking independently, writing, or cooking. These goals seem to be made in collaboration between therapists and patient. Other goals were described more generally, and on body function level, such as increasing strength and balance, indicating more therapists driven goals. Few of the discharge summaries summarized whether goals were achieved during the rehabilitation period or not. However, several did indicated goals for improvement after discharge and recommended interventions pertaining to the patient’s goals.

The therapist considered patients’ wanting further rehabilitation as an example of their motivation for further rehabilitation. However, motivation was more clearly described as the effort, or lack thereof, made by the patients themselves to improve beyond participating in therapy sessions.

*Quotes from MDT discharge summary 27, PT description*–*She has also been given equipment and instructions for self-training. She has been motivated to train and participated in both individual training and group training.*

#### The patients’ outcome and functions after stroke

Descriptions of the patients’ cognitive function and its influence on ADL were the OTs domain. While the PTs tended to provide a more general account of the patients’ cognitive abilities in terms of how they presented themselves and their medical histories, the OTs observed the patients’ cognitive challenges during a variety of ADL, such as dressing and cooking. The OTs described the patient’s memory, concentration, processing skills, and executive functions, as well as the patients’ capacity to manage multiple tasks. Additionally, to the observation OTs often included the results of Barthel ADL Index and three standardized assessment tools for cognition: MMSE, Clock Drawing Test, and Trail-Making Test.

The patients’ ability to follow instructions was mainly mentioned in instances where they were observed to be struggling with following the 3-stage command of folding a paper in the MSSE. Furthermore, the patient’s ability to learn was largely reported by the neuropsychologist, while the OTs described its effect on their ADL and interventions to address these difficulties.

*Quotes from MDT discharge summary 24, OT description–The test results [reported by the neuropsychologist] indicate reduced memory, concentration, attention, simultaneous capacity, and psychomotor pace. This can be found in complex practical activities, such as cooking. The patient has difficulty paying attention to several things at the same time. Shows difficulties with executive functions in terms of planning and implementation*. *The patient needs many repetitions for new learning as well as for learning old familiar activities, such as entering appointments to the mobile phone.*

The assessment of the sensorimotor function of the patients was mainly described by the PTs and the descriptions varied from brief reports of observed deficits to detailed accounts of neurological symptoms and their effects on ADL.

*Quotes from PT discharge summary 17* –*(the patient) Moves actively across all joints. (PT) Finding some decreased strength in the shoulder, elbows, and hip when tested against resistance. Otherwise, it is comparable to the other side. Slightly altered sensibility in the left upper and lower extremities. Normal coordination and tempo of the upper extremity. Somewhat reduced tempo in the lower extremity.*

Strength of the lower extremities and balance was often linked to descriptions of the patients’ gait quality, use of walking aids or personal support, and stair climbing. Additionally, they used mainly four assessment tools Short Physical Performance Battery was used most often, while Romberg, Clinical Test of Sensory Interaction on Balance, Timed-Up and Go, and Berg’s Balance Scale were used to a lesser degree. However, while the OTs used their assessment tools together as a package, the PTs varied in their choice of tool and the ways in which they combined them.

In the case of a patient suffering from cerebellar stroke, the PTs also observed gaze stability and its effect on the patient’s balance and dizziness. Functions of the upper extremity were described by both PTs and OTs; while the PTs often described at body function levels such as strength, tempo and coordination, the OTs linked it to challenges in ADL.

The PTs’ and OTs’ assessments of activities also included descriptions of the patients’ experience of safety, and in some instances whether the patients were following instructions when performing activities and could retain the instructions and learn of their experience.

Although both PTs’ and OTs’ interventions were sometimes listed, they were more often found to be intertwined with the assessment or summarized at the end of the assessment. However, the patients’ tolerance to intensity of the interventions was rarely mentioned, while OTs mentioned the patients’ need to balance between activity and rest. Duration and frequency of the therapy sessions were lacking. Additionally, the interventions specific focus or their effectiveness was seldom mentioned.

*Quotes from MDT discharge summary 4, PT description*–*The patient has done a lot of self-training several times a day. (PT) Have adapted dosage and intensity, as well as brought in necessary breaks.*

Improvements of the patients’ function were either expressed by the PTs in terms of better scores after retesting their function or by both the PTs and OTs in the section at the end of the MDT discharge summaries, where they described improvements observed in carrying out an activity. Furthermore, these improvements were often linked to residual challenges in functions or activities, or to recommendations for further follow-up.

All but three discharge summaries included recommendations or referrals for further physiotherapy in primary healthcare. The recommendations were primarily aimed at improving balance and strengthening the lower extremities, linking these impairments to improve walking or reducing the risk of falling. Six of the MDT discharge summaries included OTs’ recommendations for further follow-up, two clearly aimed at the patients’ needs and goals for the future, while four suggested general occupational therapeutic follow-up or the need for aids, which required the reader to deduce the specific needs that must be followed up on from previous descriptions of the patients’ function.

*Quotes from MDT discharge summary 26, OT description–She has cognitive impairments with reduced concentration, attention and executive function. This is observed to be improving during the stay [at the rehabilitation unit], but still some cognitive sequelae remain.*… *The patient is discharged to the home with an agreement for follow-up by the municipal rehabilitation team.*

## Discussion

The aim of this study was to describe and explore how PTs and OTs in hospitals document the patients’ functions and needs in their discharge summary notes. Additionally, the study aimed to describe the extent to which the discharge summaries adhered with the Norwegian stroke pathway’s must- and should criteria. Our material included 12 MDT discharge summaries including PT and OT notes, 19 PT discharge summary, but no OT discharge summaries. The findings highlight the tendency of PTs and OTs to focus on their specific areas of expertise in stroke rehabilitation in the discharge summaries. In the MDT discharge summary, PTs documented ADL and sensorimotor functions, while OTs documented ADL and cognitive functions. The PT discharge summaries from the stroke unit primarily emphasized sensorimotor function and ambulation, and while they included medical information, they omitted cognitive assessments and seldom mentioned the patients’ experiences and needs during the acute stroke rehabilitation. Furthermore, the findings demonstrate that PTs and OTs varied in their adherence to the must- and should-criteria. Although they consistently included information on ADL, they rarely addressed the must-criteria related to the patient’s motivation, ability to follow instructions, and learning. Additionally, the should-criteria regarding the intensity of rehabilitation interventions were seldom addressed in the summaries.

Our findings show that PTs and OTs prioritize descriptions of function and activity in patients with stroke, together with descriptions of interventions. This aligns with a study reporting the content of PT in the patients records [22]. Our results also support findings that show that PT and OT notes tend to be concise and emphasize documenting on body function and activity as described in ICF [21]. However, our results show little focus of the consequences for the patients’ ability to return to work or other social activities. Furthermore, while physical function and abilities were documented, cognitive assessments were omitted in the PT discharge summaries in our study. Two-thirds of patients experience some cognitive impairments after stroke [37], hence this would be considered relevant and necessary information to transfer to the subsequent therapist, whether being PT or OT.

While all the participants discharged from the rehabilitation unit were assessed by OT, we observed that neither PTs nor OTs did provide a detailed account in the MDT discharge summaries of the patients’ ability to follow instructions and learn which is important for stroke rehabilitation. However, this may indicate that the patients included in this study did not suffer from this challenge. Furthermore, the therapists used a wide range of assessment tools, which indirectly indicate that patients were capable of following instructions. This is inferred from the fact that many of these tools rely on verbal instructions that the patient must adhere to. Although it was not within the scope of our study, we did observe that neuropsychologists identified, with their assessment tools, some participants who faced difficulties in their learning abilities, which the OT did not mention. It is crucial to recognize that despite MMSE scores falling within the normal range, a significant number of stroke patients performed lower on further neuropsychological testing [38]. This indicates that patients with stroke have high risk of having hidden cognitive dysfunction which might not become obvious before experiencing more complex everyday situations.

Research has revealed that patients often become passive and feel restrained in hospital setting and they report low intensity of training in the hospital [39]. If the participants in our study have experienced this as well, it can have affected their motivation as the PTs from the stroke unit seldom described patients’ motivation in their discharge summaries. The intensity of the interventions and the patient’s tolerance to the interventions were also seldom mentioned in the included discharge summaries in our study. While the OTs mentioned the need to balance activity and rest as an intervention for some of the participants, they did not specify whether the patients experienced fatigue or how it otherwise affected the patient. Up to half of stroke patients experience fatigue [40], most often manifest during the acute stage [41]. As fatigue has a negative effect on patients’ lives, leading to both physical and cognitive difficulties, identifying and managing fatigue can greatly affect their rehabilitation potential [41]. However, as hospital settings are restrained and patients often feel passive, hidden cognitive dysfunction might not be visible before returning home [39, 42]. Nevertheless, by placing more emphasis on and documenting the level of intensity during rehabilitation, as well as the patients’ ability to tolerate intensity, both patients and therapists can gain a better understanding of fatigue and other covert cognitive functions. This increased awareness could potentially assist patients in effectively managing these symptoms and seeking assistance following their discharge.

While the MDT discharge summaries in our study often included most of the must- and should criteria, the PT discharge summaries emphasized the must- and should-criteria regarding ADL and sensorimotor functions. Which is not surprising as this is described as the PTs’ domain in the multidisciplinary rehabilitation [43]. However, they seem to distinguish between which other professionals’ information they included in their own discharge summary. They tended to include medical assessments and treatment, while they omitted the OTs’ cognitive assessments of the patient. In Norway, medical discharge summaries from hospitals are electronically transferred to the patient’s general practitioner. Allied health professions do not have access to these, unless the patient present a copy to them, as general practitioners and allied health profession in the municipalities in Norway do not share the same electronic patient record system. The allied health discharge information, on the other hand, is often available for PTs and OTs treating the patients, as they share the same patient record system. Explanations for the inclusion or omittance of other health professional information in the PT discharge summary can be several. As this study does not include the therapists’ perspectives, we can only speculate. However, the most obvious explanation could be that the PTs assumed the OTs informed the primary care themselves through an OT discharge summary, or the included participants did not have any cognitive challenges. However, stroke and rehabilitation units typically have multidisciplinary approach with regular meetings [44], which should enhance awareness of each discipline’s focus [45] and therefore should enable the PTs to address other health professions’ summaries in their discharge summaries. However, research reports that ineffective collaboration within hospitals is a hinderance to effective communication across levels of care [16]. Doctors tend not to include patients’ long-term needs [17] and multidisciplinary assessments and recommendations [18, 19]. Nurses have also reported difficulties in summarizing discharge information, but being more experienced and having templates for information transfer proved helpful in deciding which information to include [46]. To create a complete picture of the patients’ function and needs after acute stroke, international stroke guidelines recommend inclusion of multidisciplinary information in the discharge summary [2, 9, 10].

Descriptions of the patients’ involvement in rehabilitation and their understanding of stroke and rehabilitation are often omitted from their journals and discharge summaries across many health personnel [18, 47, 48]. Time, level of interaction, and quality of relationships are important factors that influence patients in terms of involvement and acquiring knowledge about stroke [16, 49–51]. While it was evident, in our study, that the patients had participated in goal setting in the rehabilitation unit because their main goals were specific to their interests, such as knitting, cooking, or independent walking, it was less visible in the PT summaries from the stroke unit. Shorter length of stay [28] and patients’ difficulties setting goals during the acute stroke phase [50] can explain the lack of focus on goal description. Another factor can be that patients sometimes leave the decision-making to the therapist as they trust their judgements [39, 52]. However, the PTs in our study did in some summaries express the patients’ agreement regarding the recommended follow-up. Setting realistic goals for treatment and experiencing improvement are crucial for patient motivation [49]. These factors are closely linked to their effort, expression of improvement, and wishes for follow-ups, as observed in our study. Therefore, the patients’ voices should be more explicitly expressed to inform the primary healthcare PTs and OTs about their motivation and involvement during rehabilitation. This will also serve as a foundation for enhancing patient involvement in primary healthcare, and thereby ensuring the continuity of what is important for the patient.

The findings in our study are consistent with another study that also noted inconsistent adherence to the same guideline and pathway, and a lack of relevant information for subsequent care in doctors’ discharge summaries [18]. The focus of hospital professionals on in-house procedures and the omission of topics relevant to follow-up care can hinder primary healthcare’s ability to ensure a seamless discharge process [45]. Hospital and primary healthcare providers often have different perspectives of the patients’ needs [53], the lack of understanding about the recipient’s information needs, skills and work pattern poses a barrier for effective communication and collaboration [45, 54, 55]. The lack of attention to the needs of the recipients can be attributed to insufficient training and reflection on the discharge process [45].

Adherence to guidelines may not solely be attributed to an inward focus but can also be influenced by the compatibility between the guidelines and the practitioners’ needs. The usefulness of research results, and thereby guidelines’ recommendations, in regards to their daily clinical practice are important for PTs and OTs [56]. While therapists acknowledge the importance of research in guiding their practices, they do not universally agree that research should be the primary determinant of their practices [57]. The selection of assessment tools, for instance, is influenced by various factors. These include the patient’s stroke outcome, the specificity of the tool to the type of therapy [58], the setting in which the tool is to be used (59), or the therapists’ familiarity with the tool [59, 60]. Furthermore, the individual practitioner’s experiences and knowledge plays a role in their adherence to guidelines [61], and experienced practitioners are an important sources of knowledge and guide clinical practice for junior practitioners [57]. The beforementioned facts indicate that the culture in the workplace can influence what is included in the discharge summary more than guidelines’ recommendations.

### Strengths and limitations

To our knowledge, only a few studies have been conducted on the assessment of PT and OT descriptions in discharge summaries for patients with stroke. Unfortunately, we were not able to collect any OT discharge summaries, although their assessments were included in the MDT discharge summaries. Since the therapists’ perspectives were not included in this study, we do not know the reason for the lack of OT discharge summaries.

On hindsight, all patients with a PT or OT discharge summary, regardless of discharge destination, could have been included in the current study. This strategy could potentially have provided more data for analysis and strengthened the findings.

Furthermore, the data for this study were collected from a single hospital. In this context, the manner of writing discharge summaries may have been affected by the hospital culture. Stroke discharge summaries in other hospitals might follow other templates and sustain a different culture that could influence their content.

Moreover, two of the authors of this paper have connections to the hospital from which the study participants were recruited. Since their affiliations had the potential to affect the study’s analysis and interpretation, the team discussed these issues and chose an analysis method that would be most suitable for addressing the research question [32]. Additionally, the first author discussed the interpretations and results with the user group, which provided additional perspectives on the study’s results. It may be argued that these precautions increase the trustworthiness of the analytical process.

A strength of the study is that all authors have experience with stroke and rehabilitation of patients. This knowledge is important in understanding and inferring the written material. However, we discussed our understanding of the discharge summaries and reached a common understanding.

## Conclusion

This study provides insights into the content of discharge summaries for patients with stroke written by hospital PTs and OTs. The therapists adhered to the Norwegian stroke pathway’s recommendation for discharge summary content in varying degrees. They clearly adhered by reporting the must-criterium ADL in conjunction with the should-criteria sensorimotor and cognitive ability function. However, they often omitted the must-criteria of describing patients’ ability to follow instructions and learn, and the should-criteria related to intensity. The patients’ voices and involvement also varied. While the PTs and OTs in the rehabilitation unit focused their intervention to obtain the patients goals, the interventions of the stroke unit’s PTs focused on the specific stroke outcome and ambulation. Paying increased attention to the elements PTs and OTs omit and understanding their influence on the continuity of care can increase the quality of their discharge summaries. To ensure the continuity of rehabilitation after hospital discharge, the PT and OT should be aware of the significant criteria for discharge summaries and should account for relevant assessments conducted by their colleagues as well as to create a complete picture of the patient’s rehabilitation needs.

## Abbreviations

PT: physiotherapists
OT: occupational therapists
ICF: disabilities, functions, and activities
MDT: multidisciplinary team
ADL: activity of daily living
MMSE: Mini mental status evaluation
SPPB: Physical performance battery
